# Urethral orifice hyaluronic acid injections: a novel animal model of bladder outlet obstruction

**DOI:** 10.1186/s12894-015-0002-0

**Published:** 2015-02-21

**Authors:** Yongquan Wang, Zhiyong Xiong, Wei Gong, Zhansong Zhou, Gensheng Lu

**Affiliations:** Center of Urology, Southwest Hospita, Third Militar, Medical University, 400038 Chongqing, China; Department of Biochemistry and Molecular Biology, College of Basic Medical Sciences, Third Military Medical University, 400038 Chongqing, China

**Keywords:** Bladder outlet obstruction, Animal model, Hyaluronic acid

## Abstract

**Background:**

We produced a novel model of bladder outlet obstruction (BOO) by periurethral injection of hyaluronic acid and compared the cystometric features, postoperative complications, and histopathological changes of that model with that of traditional open surgery.

**Methods:**

Forty female Sprague-Dawley rats were divided into three groups. Fifteen rats were subcutaneously injected with 0.2 ml hyaluronic acid at 5, 7, and 12 o’clock around the urethral orifice. Another fifteen rats underwent traditional open partial proximal urethral obstruction surgery, and 10 normal rats used as controls. After 4 weeks, filling cystometry, postoperative complications, and histopathological features were evaluated in each group. Three rats were also observed for 12 weeks after hyaluronic acid injection to evaluate the long-term effect.

**Results:**

Hyaluronic acid periurethral injection caused increased maximum cystometric capacity, maximum bladder pressure, micturition interval, and post-void residual urine volume compared with control (p < 0.01). The injection group had significantly shorter operative time, less incidence of incision infection and bladder stone formation compared with the surgery group (p < 0.01). Hematoxylin and eosin (HE) staining showed suburothelial and interstitial hyperemia edema and smooth muscle hypertrophy in both injection and surgery bladders; these were not observed in the control group. Bladder weight and thickness of smooth muscle in the injection and surgery groups were significantly greater than those in the control group (p < 0.01). Urethral epithelial hyperplasia and lamina propria inflammation were observed in the surgery group but not in the injection or control groups. Rats periurethrally injected hyaluronic acid were stable the compound was not fully absorbed in any rat after 12 weeks.

**Conclusions:**

Hyaluronic acid periurethral injection generates a simple, effective, and persistent animal model of BOO with lower complications, compared with traditional surgery.

## Background

Bladder outlet obstruction (BOO) is a common urological chronic condition in which the urine flow from the urinary bladder through the urethra is impeded. BOO can result from several diseases, including benign prostatic hyperplasia and urethral stricture in adults [1,2]. To better understand the effect of this condition on bladder structure and function, several experimental animal models have been established [3]. Those animal models that recreate BOO are critical to understanding the pathophysiology of many diseases related bladder function, and to evaluating the effects of various pharmacologic therapies.

The most widely used methodological approaches to creating BOO are the partial urethral obstruction (PUO) animal models. In these animal models, a ligature, cuff, or ring is surgically placed around the outlet of the catheterized bladder, so that when the catheter is removed, the bladder experiences increased urethral resistance [4-7]. Although animal models cannot perfectly recapitulate symptoms observed in clinic, evidence indicates that PUO rats have similar storage and micturition symptoms as human BOO patients [8-10].

However, massive trauma caused by open surgery causes some complications such as incision infection and bladder stone formation. It is also difficult to standardize the firmness of ligation, or exclude foreign material from rings in surgery PUO models. Therefore, establishing a relevant, reproducible, and minimally invasive BOO animal model would be useful.

Bulking agents have previously been injected to treat urinary incontinence in clinic. The injection seeks to increase bladder outlet resistance by oppressing the urethra and thereby reducing urinary leakage in patients with stress urinary incontinence [11]. The establishment of a BOO animal model is possible by a similar principle. Hyaluronic acid is a viscous mucopolysaccharide found in the connective tissue space. It is ideal for BOO model establishment because it is stable and histocompatible. We aimed to establish a novel BOO animal model by hyaluronic acid periurethral injection, and to compare the effects and related complications of this technique with traditional PUO surgery.

## Methods

### Animal model preparation

**Figure 1:**
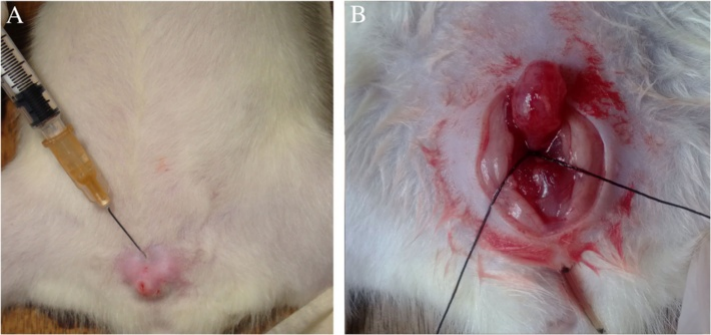
**Partial urethral obstruction (PUO) animal model. (A)** Hyaluronic acid periurethral injection **(B)** Proximal urethral ligation surgery.

All experimental protocols were approved by the Animal Research Ethics Committee of the Third Military Medical University. Forty female Sprague-Dawley rats were studied (weighing 220–260 g, Animal Center of the Third Military Medical University). All animals were anesthetized with sodium pentobarbital (50 mg/kg, i.p.) before procedures. The periurethral injection group (n = 15) underwent PUO by subcutaneous injection of 0.2 ml hyaluronic acid (Restylane, Q-Med, Uppsala,Sweden) at 5, 7, and 12 o’clock around the urethral orifice (Figure 1A). Animals in the surgery group (n = 15) underwent open surgery as previously described [12]. Briefly, a midline abdominal incision was made and the bladder and proximal urethra were dissected from the surrounding tissue. To create intravesical obstruction, a polyethylene tube (1.0 mm outer diameter) was placed beside the proximal urethra and a three-zero silk ligature was tied around the urethra and catheter. Then, the catheter was removed and the abdominal incision was closed (Figure 1B). Another ten normal rats were used as the control group. Four weeks later, mortality rates, complications, filling cystometry, and histopathological studies were evaluated in each group. To evaluate the long-term effect of hyaluronic acid in rats, another three rats were periurethrally injected with hyaluronic acid. The rats were observed for 12 weeks, at which point urethral anatomy was observed to evaluate whether or not hyaluronic acid was still present.

### Cystometric study

Four weeks after surgery or injection, cystometric studies were performed on all animals as previously described. Animals were anesthetized as described above, and the bladders were catheterized via an incision at the bladder dome with a 0.6 mm inner diameter catheter. External bladder filling was carried out using an infusion pump (3 M, Saint Paul, MN, USA) at a constant rate of 12 ml/h until micturition was detected. A cystometry catheter was connected to an external pressure transducer (RM-6240B, Chengdu, China) for the measurement of the intravesical pressure. Animals underwent cystometries by irrigation of bladders with normal saline at room temperature and the curves of intravesical pressure and frequency of micturition were recorded. In each animal, approximately 10–12 voiding cycles were recorded and then the mean of the voiding cycles was calculated. The following parameters were evaluated: maximum cystometric capacity (MCC), maximal micturition pressure (Pmax), frequency (micturition interval), post-voiding residual volume (PRV), and detrusor instable contraction (DI). DI was defined as the significant non-voiding detrusor contractions (NVCs) higher than 10 cmH_2_O.

### Histopathological study

Four weeks after operation or injection, the animals were sacrificed and the bladders in each group were excised en bloc with the urethra. Bladders were weighed, and the ratio of bladder weight (mg) to body weight (g) was calculated in each surviving animal. Specimens of kidney, ureter, bladder, and urethra from each of the groups were immediately fixed with 10% formalin. After fixation, the tissues were embedded in paraffin, and 5-μm-thick tissue sections were cut. All of the specimens were stained using hematoxylin and eosin (HE) and viewed under a light microscope to evaluate histopathological changes. The thickness of the smooth muscle layer between the serosa and submucosa of the bladder dome were measured and compared among the three groups. All histopathological findings were determined by a pathologist in a blinded fashion.

### Statistical analysis

Analysis was performed by SPSS11.0. All data were analyzed for normality of distribution utilizing the Kolmogorov-Smimov test and presented as the mean ± SEM. Data were subjected to t-test. For all statistical tests, P < 0.05 was considered significant.

## Results

All animals in the injection and control groups survived until the day of evaluation. One of the animals in the surgery group died because of severe incision infection and bladder stones. The average operative time of injection was 3.5 ± 0.3 minutes, which was significantly shorter than that in the surgery group with 15.6 ± 5.4 minutes (p < 0.01).

**Figure 2:**
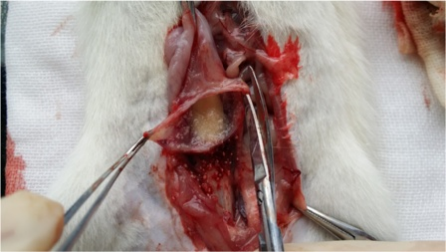
**Bladder stone formation in surgery animal after 4 weeks of ligation.**

Two animals had incision infections and bladder stone formation (Figure 2) in the surgery group. There were no complications in the injection or control groups (Table 1). All animals with complications were excluded from the cystometric study.

**Table 1:** **Post-operative complications in injection, surgery, and control groups**

**Groups**	**Mortality**	**Incision infection**	**Bladder stone**
Injection group N = 15	0	0	0
Surgery group N = 15	1	2	2
Control group N = 10	0	0	0

**Figure 3:**
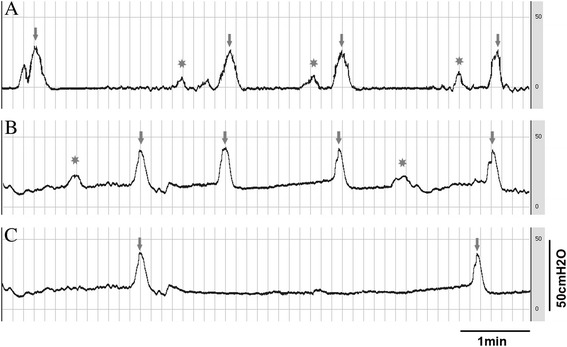
**Filling cystometric curves in injection, operation, and control rats. (A)** and **(B)** show respective injection and surgery bladders with significant decreases in micturition interval and frequent non-voiding contractions (NVCs) in the storage phase. **(C)** Control bladder displayed micturition frequency and good bladder compliance. *Non-voiding contractions (NVCs); ↓: Micturition.

Filling cystometric data showed that both the injection and surgery procedures caused increased MCC, Pmax, frequency, and PRV compared with the control (p < 0.01) (Table 2). Both methods of obstruction caused cystometric changes in the bladder consistent with detrusor overactivity, which showed increasing spontaneous activity displayed as non-voiding contractions (NVCs) greater than 10 cmH_2_O during the bladder storage period (Figure 3).

**Table 2:** **Filling cystometry results in injection, operation, and control groups**

**Groups MCC (ml) Micturation interval (s)**		**Pmax (cmH**_**2**_ **O)**		**PRV (ml)**	**NVC** ^**#**^
Injection group N = 15	1.66 ± 0.17^*^	39.47 ± 3.13^*^	152.13 ± 20.13^*^	0.26 ± 0.10^*^	3.21 ± 0.42^*^
Surgery group 0.28 ± 0.13^*^ N = 12	1.81 ± 0.59^*^	42.11 ± 7.99^*^	165.23 ± 56.30^*^		2.85 ± 0.67^*^
Control group N = 10	1.38 ± 0.13	31.28 ± 3.37	256.2 ± 25.55	0	0

Both injection and surgery groups caused typical obstruction characteristics with increasing MCC, Pmax, frequency, and PRV compared with the control group. The data are shown as mean value ± SD.

*p < 0.05 compared to control group.

^#^the frequency of NVC was calculated in every 10 minutes.

MCC, maximum cystometric capacity; Pmax maximum bladder pressure during micturition; PRV, post-void residual urine volume.

After the animals were sacrificed, gross and microscopic anatomical changes of the organs were examined. There was no significant difference in body weight among the control (209.2 ± 16.2 g), surgery (210.2 ± 14.6 g), and injection (206.1 ± 17.3 g) rats at 4 weeks. However, the bladder weights in the surgery (3.1 ± 0.6 g) and injection (2.6 ± 0.3 g) groups were significantly greater than those in the control group (0.9 ± 0.1 g). The ratio of bladder weight (mg) to body weight (g) was used to evaluate bladder hypertrophy. The ratio increased in both the surgery (14.1 ± 3.7) and injection groups (12.6 ± 1.3) compared with the control group (4.7 ± 0.3, p < 0.01). There was a significant increase in the thickness of smooth muscle in the bladders of the injection (805 ± 77 μm) and surgery groups (961 ± 186 μm) compared with the control group (442 ± 39 μm, p < 0.01).

**Figure 4:**
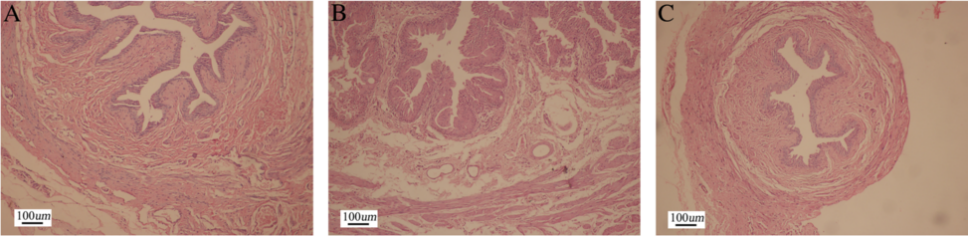
**Histological characteristics of injection, operation, and control bladders.** There was a significant increase in the thickness of smooth muscle in the bladders of the injection **(A)** and surgery groups **(B)** compared with the control group **(C)**. Suburothelium and interstitial hyperemia edema and smooth muscle hypertrophy were found in both injection and surgery bladders. Severe suburothelium inflammation and hyperemia were observed in the surgery group but not in the injection group.

**Figure 5:**
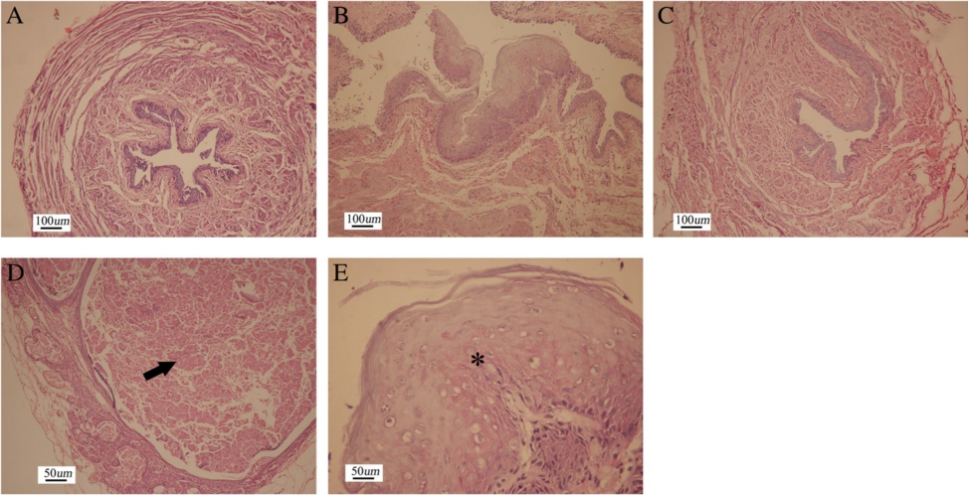
**Histological characteristics of injection, operation, and control proximal urethras.** The proximal urethra in injection rats showed mild smooth muscle hypertrophy without epithelial proliferation and suburothelium inflammation **(A)** similar to the control **(C)**. Epithelial proliferation and inflammation were seen around the urethra ligation in the surgery group **(B)**. Injected hyaluronic acid is indicated with an arrow **(D)**. Squamous metaplasia because of severe epithelial proliferation and inflammation is indicated with an asterisk in the surgery group **(E)**.

HE staining showed that all rats in three groups had normal kidneys and ureters. Examination of the bladder wall revealed an enlarged and hypertrophied bladder in each rat in the surgery and injection groups. Suburothelium inflammatory changes were observed in all surgery rats but not in those rats in the injection group (Figure 4A and B). In the proximal urethra, signs of epithelial proliferation and inflammation were observed around the urethra ligation in all surgery rats (Figure 5B). In some rats (3 of the 12 rats), severe epithelial proliferation caused squamous metaplasia near the ligation in the surgery group (Figure 5E). Unlike the surgery ligation rats, the urethra in the injection rats showed mild smooth muscle hypertrophy without epithelial proliferation and suburothelium inflammation (Figure 5A). Injected hyaluronic acid can be seen as a red dyed zone with a clear boundary, which oppressed the surrounding tissue without any inflammatory signs (Figure 5D). Histological study of the rats in the control group did not reveal any abnormalities (Figures 4C and 5C).

**Figure 6:**
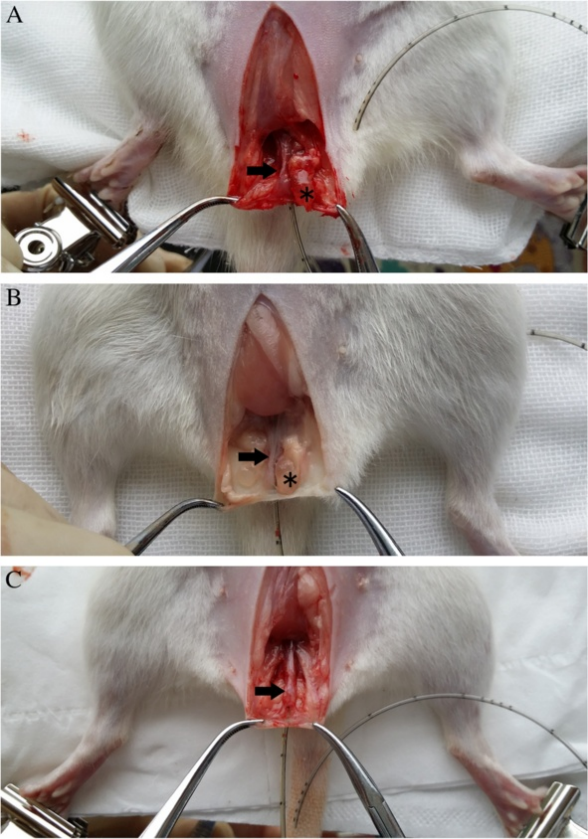
**Urethral anatomy after hyaluronic acid injection for 4 and 12 weeks.** Injected gel hyaluronic acid stably existed around the urethra. The size of the residual hyaluronic acid was not different from 4 weeks **(A)** to 12 weeks **(B)**. Normal rat urethral anatomy was also performed as control **(C)**. Hyaluronic acid is indicated with an asterisk, and the urethra is indicated with an arrow.

After 12 weeks, three hyaluronic acid injected rats were sacrificed and urethral anatomy was observed. The semitransparent hyaluronic acid gel was still present around the urethra (Figure 6) in all three rats. The size of the residual hyaluronic acid was the same between the 4 and 12 week rats. Therefore, there was no significant absorption of hyaluronic acid after 12 weeks.

## Discussion

The partial bladder neck ligation model is widely used in many studies on BOO, but the procedure is complex and there is a high risk of complications. Operative time and the degree of ligation are difficult to standardize, and severe inflammation around the urethral ligation can often result in excessive obstruction. Further, the fibrous scar in the abdominal skin and muscle, which does not occur in patients with BOO, may affect the accuracy studies using this model [13]. Placing a metal ring loosely around the proximal urethra has been used to create BOO models [7,14]. However, rejection of the foreign material is difficult to avoid.

Using bulking agents to increase bladder outlet resistance has been used in urinary incontinence treatment for some human patients. However, clinical case reports indicate that periurethral polytetrafluoroethylene (Teflon) injection can cause BOO in some patients [15].

Hyaluronic acid is an anionic, nonsulfated glycosaminoglycan that is distributed widely throughout connective, epithelial, and neural tissues. It is popular for filling soft tissue defects such as facial wrinkles or lip augmentation in plastic surgery because it is effective, easy to administer, and safe. Some urologists have attempted to use hyaluronic acid injection to treat vesicoureteral reflux in children [16-18]. Hyaluronic acid is biocompatible and can be exist stably in the body, so it could be used to observe long-term pathophysiological changes in some chronic diseases. Clinical trials indicate that hyaluronic acid can persist up to 6–18 after injection in humans [19]. Therefore, hyaluronic acid appears to be a perfect agent to creating and simulating urinary obstruction. However, no reported BOO model has been produced by subcutaneous injection of hyaluronic acid.

In this study, we used hyaluronic acid injection to produce BOO in rats. Using this method, no open surgery or ligation was needed, greatly simplifying the operational process. Therefore, the procedure is safer and more stable compared with traditional surgery. Moreover, the degree of obstruction was easier to control with hyaluronic acid injection. Filling cystometry studies showed that periurethral injection of hyaluronic acid could achieve the same bladder outlet obstructive effects as open surgery. The detrusor overactivity presenting of NVCs appeared in both the injection and surgery bladders. Furthermore, the observed complication rate in the surgery group was higher than that in the injection group. In histological observation, both injection and surgery bladders showed suburothelial and interstitial hyperemia edema and smooth muscle hypertrophy, which are the compensatory responses after urinary tract obstruction. However, significant differences in the bladder and urethra were observed between the surgery and injection groups. In the rats with a urethral ligation, the bladder and urethra showed signs of fibrosis, inflammation, and muscular hypertrophy. The rats with injection had only mild muscular hypertrophy without epithelial proliferation or lamina propria inflammation. Therefore, the injection of hyaluronic acid seems to cause only some physiological changes in muscular thickening of the bladder wall and increased bladder weight, and minimizes the interference of inflammation. These symptoms more closely mimic those observed of BOO in clinic. To further evaluate the long-term homeostasis of hyaluronic acid, we observed urethral anatomy and found that injected hyaluronic acid was still present after 12 weeks. This suggests that the BOO model is effective for longer examinations. The cystometry data in the injection group had a smaller standard deviation than that in the surgical group, which indicated that the model is more stable and repeatable.

While hyaluronic acid is expensive, the amount necessary for injection in rats is small. Considering that it significantly reduced the amount of animals and time required for study, we believe that it is more cost-effective than the traditional surgery model.

## Conclusions

We demonstrated that periurethral injection of hyaluronic acid creates a relatively simple, effective, and persistent animal model of BOO that has fewer complications than that of the traditional surgery model. The model is simple to establish and provides consistent pathophysiological changes of BOO that will be useful for future study.

## Abbreviations

BOO: Bladder outlet obstruction
PUO: Partial urethral obstruction
MCC: Maximum cystometric capacity
Pmax: Maximal micturition pressure
PRV: Post-voiding residual volume
DI: Detrusor instable contraction
NVCs: Non-voiding detrusor contractions
HE: Hematoxylin and eosin
